# Effectiveness and durability of darunavir/ritonavir (DRV/r) in DRV/r-experienced HIV-1-infected patients in routine clinical practice

**DOI:** 10.7448/IAS.17.4.19786

**Published:** 2014-11-02

**Authors:** Andrea Antinori, Massimo Galli, Nicola Gianotti, Cristina Mussini, Tiziana Quirino, Katia Sterrantino, Daniela Mancusi, Roberta Termini

**Affiliations:** 1 Clinical Department, National Institute for Infectious Diseases L. Spallanzani, Rome, Italy; 2 Department of Infectious Disease, L. Sacco University Hospital, Milan, Italy; 3 Clinic of Infectious Diseases, San Raffaele Hospital, Milan, Italy; 4 Institute of Infectious Diseases, University of Modena and Reggio Emilia, Modena, Italy; 5 Unit of Infectious Diseases, Busto Arsizio Hospital, Busto Arsizio, Italy; 6 Division of Tropical and Infectious Diseases, Careggi Hospital, Florence, Italy; 7 Medical Affairs, Janssen SpA, Cologno Monzese, Italy

## Abstract

**Introduction:**

This was a descriptive non-interventional study in HIV-1-infected patients treated with DRV/r conducted in the clinical setting, with a single-arm prospective design. The primary objective was to collect data on utilization of darunavir/ritonavir (DRV/r) under the conditions described in the marketing authorization. Efficacy (measured as viral load [VL] <50 copies/mL and CD4+ cell count) was evaluated for DRV/r in combination with other antiretroviral (ARV) agents in routine clinical practice in Italy.

**Materials and Methods:**

Here we describe an analysis of effectiveness and durability data from two cohorts of DRV/r-experienced patients with HIV-1 infection, already receiving DRV/r according to usual clinical practice, collected prospectively from June 2009 to December 2012: Cohort 1, data from patients from the DRV/r Early Access Program (TMC114-C226 study; N=235 patients) and Cohort 2, a separate cohort of ARV-DRV/r-experienced patients (N=407 patients), treated with DRV/r in the market. Patient characteristics are shown in Table 1.

**Results:**

The median length of DRV/r exposure during the study was 925 days (interquartile range [IQR] 692–1006) in Cohort 1, and 581 (IQR 508–734) days in Cohort 2. Of those patients that completed the study, 94% and 87% of patients were virologically suppressed in Cohort 1 and 2, respectively, at last study visit (LSV). As expected, the virological suppression rate was higher in patients with baseline VL <50 copies/mL (Table 2). Mean CD4+ cell counts improved from baseline to LSV in both cohorts (Cohort 1: +54 cells/µL [95% CI 31, 77] and Cohort 2: +59 cells/µL [95% CI 44, 73]). High persistence rates were seen in both cohorts, with 75.3% of patients in Cohort 1 and 82.6% in Cohort 2 remaining on treatment at LSV; very few patients discontinued due to virologic failure (Table 1). Other reasons for study discontinuation are shown in Table 1. Very few patients changed DRV/r dosing during the study, 15 from 1200 to 800 mg o.d.

**Conclusions:**

In patients already treated with DRV/r, DRV/r-based ARV treatment provided effective viral suppression with long-lasting durability, low virological response failure, low discontinuation rates and good tolerability. These data confirm DRV/r to be an effective treatment choice in previously treated patients.

**Table 1:** Patient characteristics

Baseline characteristics Parameters	Cohort 1 (N=235) N, (%)	Cohort 2 (N=407) N, (%)
Age, yrs, mean±SD	49.3±7.1	46.6±9.4
Female, n (%)	45 (19.1)	107 (26.3)
VL (HIV-RNA) <50 copies/mL, n (%)^a^ at study enrolment	192 (85)	298 (74.9)
CD4+<100 cells/µL, n (%)^b^ at study enrolment	7 (3.1)	25 (6.2)
Duration of HIV infection, n (%)		
0–1 year	0	53 (13.0)
>1–10 years	6 (2.6)	82 (20.1)
>10–15 years	62 (26.4)	66 (16.2)
>15–20 years	89 (37.9)	85 (20.9)
>20 years	76 (32.3)	110 (27.0)
NA	2 (0.9)	11 (2.7)
CDC clinical stage, n (%)		
A	30 (12.8)	139 (34.2)
B	80 (34.0)	121 (29.7)
C	125 (53.2)	147 (36.1)
Time since first DRV dose (days), at study enrolment – mean±SD	1242±208	501±402
Study Duration, days – mean±SD	812±286	583±188
DRV dose at study entry, mg/day, n (%)		
1200	232	262
800	3	145

a VL data only available in 226 Cohort 1 and 398 Cohort 2 patients.

b CD4+ data only available in 226 Cohort 1 and 403 Cohort 2 patients.

**Table 2:** Patient characteristics

Virological EfficacyParameters	Cohort 1 (N=235) N, (%)	Cohort 2 (N=407) N, (%)
LSV VL [HIV-RNA <50 copies/mL], n (%)^a^		
All	203 (88.6)	331 (83.6)
BL VL <50 copies/mL	177 (94.1)	263 (89.2)
BL VL ≥50 copies/mL	19 (59.4)	60 (65.2)
No values for BL VL	7 (77.8)	8 (88.9)
Total discontinuations at LSV, n (%)	56 (24.7)	71 (17.4)
Reasons for discontinuation, n (%)		
Insufficient virological response	8 (3.4)	4 (1.0)
Death	10 (4.3)	9 (2.2)
Study emergent adverse event	3 (1.3)	9 (2.2)
Other study emergent medical reason	0	1 (0.2)
Lack of compliance	4 (1.7)	8 (2.0)
Lost to follow-up	23 (9.8)	18 (4.4)
Other	9 (3.8)	20 (4.9)

a VL data only available in 226 Cohort 1 and 398 Cohort 2 patients.

